# Evaluation of a patient self-medication program in allogeneic hematopoietic stem cell transplantation

**DOI:** 10.1177/10781552211043525

**Published:** 2021-09-27

**Authors:** Samantha Polito, Lina Ho, Ian Pang, Celina Dara, Auro Viswabandya

**Affiliations:** 7989 University Health Network, Research Ethics Board, Toronto, ON, Canada

**Keywords:** self medication program, allogeneic hematopoietic stem cell transplantation, allogeneic bone marrow transplantation, patient self medication program, medication program evaluation

## Abstract

**Introduction:**

Patients admitted for allogeneic hematopoietic stem cell transplantation (allo-HSCT) are discharged with multiple new medications. At our institution, a new patient Self Medication Program (SMP) was implemented on the allo-HSCT units. An SMP allows patients to practice self-administration of medications in a controlled environment before discharge. We assessed the impact of the SMP on patient medication knowledge, self-efficacy, adherence, and safety. Patient and staff satisfaction with the SMP was also explored.

**Methods:**

Participants in the SMP group received medication counseling by a pharmacist and self-managed their medications with nursing supervision until discharge. Participants in the pre-SMP group received medication counseling by a pharmacist at discharge. All participants completed a Medication Knowledge and Self-Efficacy Questionnaire before discharge and at follow-up. Safety endpoints were assessed for SMP participants.

**Results:**

Twenty-six patients in the pre-SMP group and 25 patients in the SMP group completed both questionnaires. Median knowledge scores in the pre-SMP group versus the SMP group were 8.5/10 versus 10/10 at discharge (*p* = 0.0023) and 9/10 versus 10/10 at follow-up (*p* = 0.047). Median self-efficacy scores were 38/39 in the pre-SMP group versus 39/39 in the SMP group at both discharge and follow-up (*p*_discharge_ = 0.11, *p*_follow-up_ = 0.10). The SMP was associated with at least 1 medication event in 7 participants, but no medication incidents. Patient and staff surveys showed a positive perceived value of the SMP.

**Conclusion:**

Our results demonstrate that the SMP is associated with durable, improved medication knowledge, a trend towards improved self-efficacy, and largely positive perceptions among both staff and patient participants.

## Introduction

Recipients of an allogeneic hematopoietic stem cell transplantation (allo-HSCT) are discharged on multiple new medications. Nonadherence to these medications can cause severe adverse effects such as myelosuppression and organ dysfunction, and lead to poor health outcomes such as infections, graft rejection, graft-versus-host disease (GVHD), and hospital readmissions. Some patients may already be on multiple medications prior to their transplant due to pre-existing comorbidities, and this can make their medication regimen even more complex and confusing. For this reason, medication knowledge and adherence are particularly vital in this patient population. Evidence from the community pharmacy setting has linked patient medication knowledge to adherence, highlighting the importance of successful medication education.^1,2^

## Background

The previous standard of care for discharge medication education on the allo-HSCT units at the Princess Margaret Cancer Center (PM), a part of the University Health Network (UHN), was a detailed one-on-one counselling session performed by a pharmacist within 24 hours before discharge. In addition to counselling, patients were provided with a personalized medication chart detailing their discharge medication schedule. A new patient Self Medication Program (SMP) was recently implemented on the allo-HSCT units at PM. An SMP provides patients with an opportunity to practice self-administration of their medications in a safe and controlled environment. Medication counseling and medication charts are provided to patients earlier during their hospital stay at a time determined by the interprofessional health care team. Medications are provided to patients in labeled medication vials in a fashion similar to how they would be dispensed from a retail pharmacy, rather than as individually unit dosed. The patient is then given autonomy, with supervision, in administering their medications as they demonstrate competence in doing so. Patients self-administer each dose of their medications in the presence of their nurse, who verifies the dose and then documents that the dose has been taken by signing off on the patient’s electronic Medication Administration Record (eMAR). This ensures that the patient is taking the correct dose at the correct time and that each dose is documented as per hospital standards.

Evidence supporting the efficacy of an SMP exists in a multitude of patient populations, both in the acute care and rehabilitation settings.^3–5^ In the geriatric rehabilitation population, one randomized controlled trial found that implementation of an SMP did not affect patient morale, medication knowledge, or ability to self-medicate on discharge; however, medication errors at 1-month follow-up were significantly decreased.^3^ Conversely, a study in the multi-organ transplant population found that an SMP did improve patient medication knowledge in the areas of drug identification, indication, and dose, but had no effect on knowledge of medication side effects.^4^ In terms of acceptability of an SMP, the results were mixed. One study in the acute care setting found that patients ‘found the program worthwhile’; interestingly, another study in the rehabilitation setting described patient reluctance to participate in an SMP.^5,6^ The explanation cited for this hesitancy was the potential involvement of the ‘sick role’, in which patients feel that when they are institutionalized, they are ill and unable to be involved in their own care.^5,7^ Historically, feedback from staff involved in the implementation of an SMP was positive, noting that the benefits (such as increased patient familiarity with medications, and increased patient safety after discharge) generally outweighed the difficulties (such as increased workload, and time constraints).^5,8^ Common themes in the existing literature evaluating an SMP include the importance of endpoints demonstrated to predict meaningful patient outcomes, pre- and post-testing in order to avoid quasi-experimental design, and sufficient time between intervention and follow-up to accurately measure effect.^9^ Notably missing from the literature is evidence from the allo-HSCT patient population.

## Methodology

This is a prospective pre- and post-cohort comparison between study participants who have completed the SMP prior to discharge from the hospital and those who have received conventional discharge medication education. Participants included individuals who were admitted as inpatients and received an allo-HSCT between December 1, 2017 and August 10, 2018. Participants admitted for a second allo-HSCT due to graft failure or disease relapse were excluded. Participants must also have been planned for discharge to the community (i.e. not transferred to another acute care or long-term care institution) during the same hospital visit. The date of hospital discharge for participants in the pre-SMP group must have pre-dated the implementation of the SMP. Participants in the SMP group must have completed the SMP. Participants who were unable to complete the SMP or questionnaire due to cognitive or language barriers, as identified by a study team member or delegate, were excluded. All study participants provided written informed consent prior to study enrollment.

The primary study objective was to assess the impact of the SMP on patient medication knowledge, self-efficacy, and adherence, and to determine whether the impact was maintained over time. The secondary objective was to assess patient safety during the implementation of the SMP by recording the number and nature of reminders and corrective actions made by nurses, and the number and nature of medication incidents reported. The tertiary objective was to evaluate the acceptability of the SMP by assessing patient and staff perceptions of, and satisfaction with, the program. We hypothesized that patients who completed the SMP would demonstrate better medication knowledge and medication self-efficacy at both discharge and follow-up, and have better medication adherence at follow-up, versus patients who had received the previous standard of care. We also hypothesized that while nurse interventions such as medication administration reminders and corrections would be necessary for some patients on the SMP, it would not result in an increased occurrence of medication incidents such as missed or incorrectly taken doses. Finally, we hypothesized that patient perceptions of the program would be positive and that while staff may perceive an initial increase in workload during the implementation of the program, ultimately the perceived value of the program would outweigh this.

### Primary efficacy endpoints

To determine the efficacy of the SMP compared to the previous standard of care, a medication knowledge and self-efficacy questionnaire were administered to subjects at two time points: (1) within 24 h prior to discharge (questionnaire 1; see Appendix  1) and (2) at a follow-up appointment 3–5 weeks post-discharge (questionnaire 2; see Appendix 2). The questionnaire scores in the pre-SMP group and the SMP group were compared.

The first section of the questionnaires includes knowledge-based questions targeted to the allo-HSCT patient population that was developed by the study team based on 5 medication competencies.^1,10^ The 5 medication competencies are medication name, indication for the medication, how and when to take the medication, important side effects, and what to do if a dose is missed. The second section of the questionnaires includes a modified version of the Self-Efficacy for Appropriate Medication Use Scale (SEAMS; see Appendix 3), which has previously been validated,^11^ is generalizable to a multitude of patient populations and is easy to use.^12^ With permission from the original authors, the scale was modified by the study team to tailor the scale elements to the study population, while retaining the integrity of the scale. In questionnaire 2, a self-reported visual analog scale rating is included to measure medication adherence since discharge.

### Secondary safety endpoints

Secondary endpoints included the number and nature of medication events (defined as any nurse-initiated reminders to take medications or corrections to medication doses and/or timing), as well as the number and nature of medication incidents (defined as any medication events that were not corrected prior to reaching the patient), in the SMP group. These endpoints were collected through a review of the eMAR. Through the review of the hospital’s electronic patient record, the number and nature of readmissions during the follow-up period were also recorded.

### Tertiary endpoints

In order to evaluate patient and staff perceptions of, and satisfaction with, the SMP, both a patient and staff survey were administered (Appendices 4 and 5, respectively). The staff survey was computer-based, and emailed out to all PM staff involved in the implementation and/or administration of the SMP on the allo-HSCT units (nurses, nurse practitioners, pharmacists, and physicians; approximately 80 individuals in total) approximately 8 months after implementation of the SMP. The patient survey was paper-based, and administered to a cohort of 20 patients (different from those who were part of the SMP group described above) who had completed the SMP during their hospital stay. Surveys were administered by a study team member or delegate not directly involved in the care of survey respondents while they were in hospital.

### Statistical analysis

The results of the medication knowledge and self-efficacy questionnaires were compared using nonparametric statistical tests. The questionnaires produced 2 scores per participant: medication-taking knowledge and self-efficacy. Differences in scores at discharge and at 3–5 week follow-up between study groups were analyzed using the Mann–Whitney *U* test. Difference in readmission rates between study groups was assessed using a Chi-square test. Participant baseline characteristics were described using descriptive statistics (median, interquartile range (IQR)).

## Results

**Figure 1.**
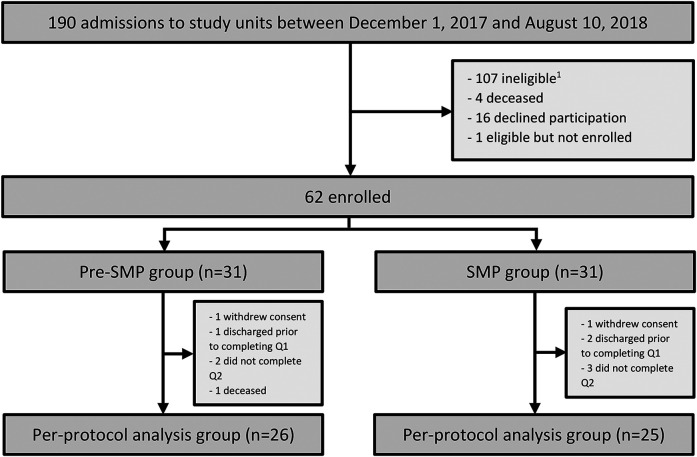
Study flow diagram. Q1  =  questionnaire 1. Q2 = questionnaire 2. ^1^Reasons for study ineligibility included admission for reason other than an allo-HSCT, admission for a second allo-HSCT, planned discharge to another acute care facility, or significant cognitive or language barrier identified by a member of the study team or delegate. allo-HSCT: allogeneic hematopoietic stem cell transplantation.

There were 190 admissions to study units during the study period. Of these admissions, 62 participants were enrolled in the study (Figure 1). The most common reasons for not enrolling in the study included failure to meet inclusion criteria (107/128, 84%) and declining participation (16/128, 13%). Thirty-one participants were enrolled in each of the pre-SMP and SMP groups, making up the intention-to-treat population. Twenty-six participants in the pre-SMP group and 25 participants in the SMP group completed both questionnaires, making up the per-protocol population.

### Baseline characteristics

Participants in the per-protocol population were evenly matched. In both groups, the majority of participants were male (69% in the pre-SMP group, 72% in the SMP group), <65 years old (69% in the pre-SMP group, 76% in the SMP group), and had a diagnosis of acute leukemia (65% in the pre-SMP group, 56% in the SMP group). The median number of medication changes at discharge, defined as any new, discontinued, adjusted, or held medication, was 8 in both groups (IQR_pre-SMP_  =  3.5, IQR_SMP_  =  3, *p*  =  0.64). Additional information about participants in the per-protocol population is presented in Table 1.

**Table 1.** Baseline characteristics.

	Pre-SMP (*n* = 26)	SMP (*n* = 25)
Gender, *n* (%)		
Male	18 (69)	18 (72)
Female	8 (31)	7 (28)
Age, years, *n* (%)		
<65	18 (69)	19 (76)
≥65	8 (31)	6 (24)
Highest level of education completed, *n* (%)		
Grade school	0 (0)	1 (4)
High school	5 (19)	10 (40)
Post-secondary	21 (81)	13 (52)
Preferred not to disclose	0 (0)	1 (4)
Primary diagnosis, *n* (%)		
Acute leukemia	17 (65)	14 (56)
Other	9 (35)	11 (44)
Median # of medication changes* at discharge (IQR)	8 (3.5)	8 (3)
	*p* = 0.64	

SMP: Self Medication Program; IQR: interquartile range.

### Efficacy endpoints

**Figure 2.**
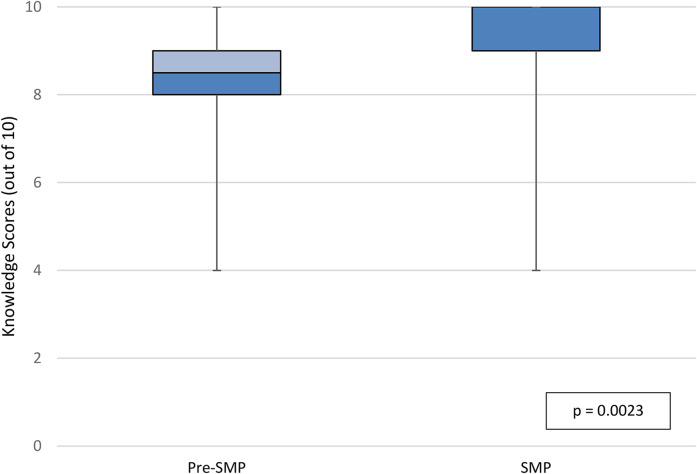
Knowledge scores at discharge, per-protocol population.

**Figure 3.**
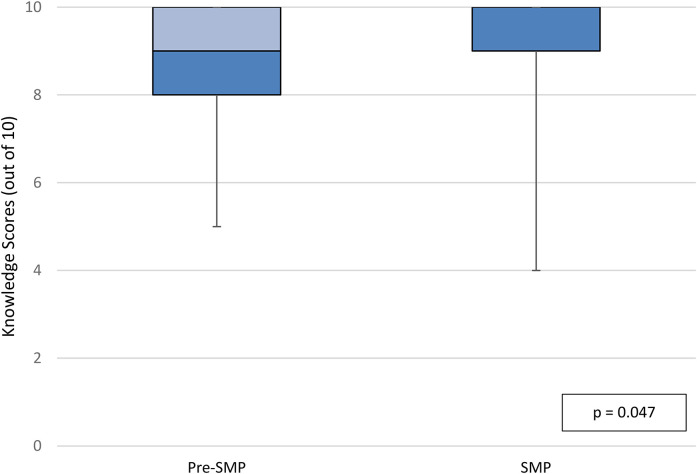
Knowledge scores at follow-up, per-protocol population.

In the per-protocol population, median knowledge scores at discharge were 8.5 out of 10 in the pre-SMP group versus 10 out of 10 in the SMP group (IQR_pre-SMP_  =  2, IQR_SMP_  =  1, *p*  =  0.0023; Figure 2). Median knowledge scores at follow-up in the per-protocol population were 9 out of 10 in the pre-SMP group versus 10 out of 10 in the SMP group (IQR_pre-SMP_  =  2, IQR_SMP_  =  1, *p*  =  0.047; Figure 3). Within groups, there was no statistically significant difference in knowledge scores at discharge versus at follow-up. Participants in the pre-SMP group most frequently provided incorrect responses to knowledge questions regarding what to do if a dose of medication is missed at discharge and medication indication at follow-up. In the SMP group, the domain of knowledge question that participants most frequently answered incorrectly at both discharge and follow-up was important side effects. Additional details regarding the distribution of incorrect responses to knowledge-based questions can be found in Appendix 6A.

**Figure 4.**
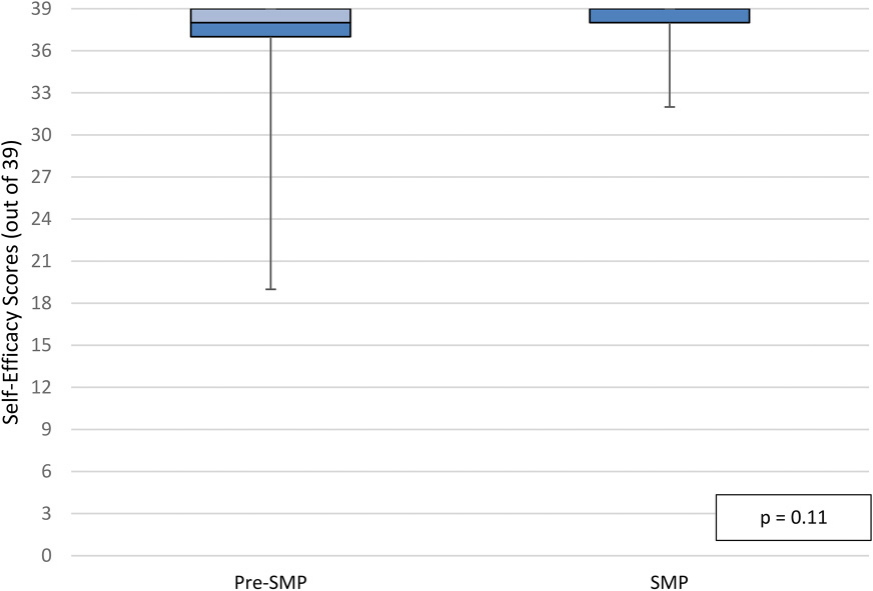
Self-efficacy scores at discharge, per-protocol population.

**Figure 5.**
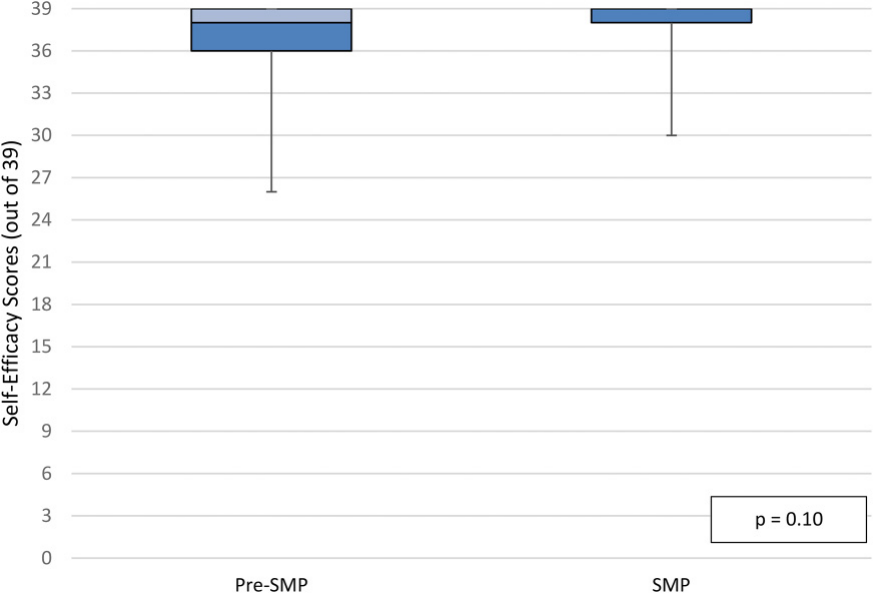
Self-efficacy scores at follow-up, per-protocol population.

**Figure 6.**
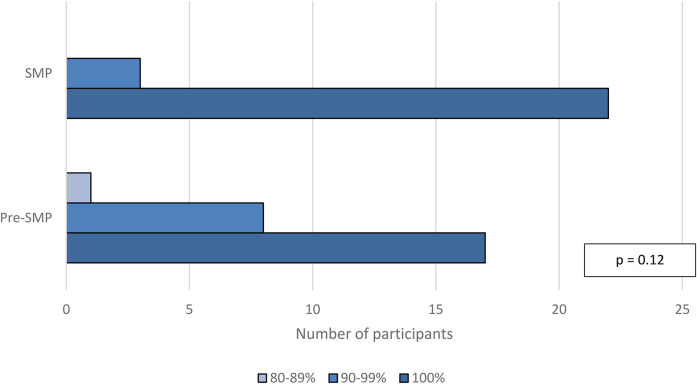
Self-reported adherence scores at follow-up, per-protocol population.

Median self-efficacy scores in the per-protocol population at discharge were 38 out of 39 in the pre-SMP group versus 39 out of 39 in the SMP group (IQR_pre-SMP_  =  2, IQR_SMP_  =  1, *p*  =  0.11; Figure 4). Median self-efficacy scores in the per-protocol population at follow-up were also 38 out of 39 in the pre-SMP group versus 39 out of 39 in the SMP group (IQR_pre-SMP_  =  3, IQR_SMP_  =  1, *p*  =  0.10; Figure 5). Additional data for participant responses for self-efficacy questions is illustrated in Appendix 6B. There was no statistically significant difference in self-efficacy scores at discharge and follow-up within groups. Finally, median self-reported medication adherence scores in the per-protocol population at follow-up were 100% in both groups (IQR_pre-SMP_  =  5, IQR_SMP_  =  0, *p*  =  0.12; Figure 6).

### Safety endpoints

The SMP was associated with at least 1 medication event in 7 participants; none of which led to a medication incident. The reported medication events were: medication administration reminders (5 events in 4 participants), wrong medication (1 event), wrong dose (3 events), and wrong administration time (1 event).

Eight participants (26%) in the pre-SMP group and 6 participants (19%) in the SMP group experienced at least one hospital readmission during the follow-up period (*p*  =  0.54); one participant in the pre-SMP group experienced 2 hospital readmissions. The reasons for hospital readmission in the pre-SMP group included: documented infection (2 events), poor oral intake (3 events, 2 in the same participant), GVHD (3 events), and cytomegalovirus viremia (1 event). In the SMP group, reasons for hospital readmission included: GVHD (1 event), febrile neutropenia of unknown source (2 events), fever without neutropenia (1 event), central venous catheter infection (1 event), and generalized weakness (1 event).

### Patient and staff acceptability

A patient satisfaction survey was administered to 20 patients. Of these patients, 19 completed the survey; one patient left the survey blank except for one comment, stating that they found the process ‘overwhelming’, and expressed a desire for medications to be provided in blister packs. None of the patients reported ever participating in an SMP in the past. In terms of the amount of information received about the program, results were positive: 19/19 patients reported that the amount of information they received prior to starting the program was just right, while 18/19 felt the same with the amount of information they received during the program (one patient commented that medication vials were not labeled with enough information). A 7-point Likert scale was used to gauge patient opinions. These results were also positive, with most patients agreeing with statements that indicate the following: clear language used (18/19 agree or strongly agree), clear instructions used (19/19 agree or strongly agree), medication grid easy to follow (18/19 agree or strongly agree), sense of safety (19/19 agree or strongly agree), sense of increased medication knowledge (17/19 agree or strongly agree, 1/19 somewhat agree) including how and when to take them (18/19 agree or strongly agree, 1/19 somewhat agree), and increased confidence in the ability to manage changes to medications (17/19 agree or strongly agree, 1/19 strongly disagree). One patient reported requiring assistance with medications after discharge by using a pill organizer. Patients were also asked to provide free form comments about their positive and negative opinions of the SMP. General themes for positive aspects of the program included increased confidence, a positive value of practice with medication management, and increased preparedness for transition to home. General themes for the negative aspect of the program included requests for additional drug information, specifically more information on medication side effects and drug interactions.

Twenty-two staff members responded to the staff acceptability survey (15 nurses, 4 pharmacists, 2 physicians, and 1 nurse practitioner). None of the survey respondents had prior experience with an SMP. Interestingly, while only 6/22 respondents felt that their workload had increased as a result of the SMP, 12/22 respondents reported experiencing difficulties related to the program, citing patients’ reluctance to participate, frequent medication and dose changes, and frequent reinforcement of teaching as common problems. A 7-point Likert scale was used to gauge staff opinions on the impact of the SMP. Results indicated that staff members felt that the SMP had a positive impact, with all respondents indicating agreement that the SMP increased patients’ medication knowledge (12/22 agree or strongly agree, 5/22 somewhat agree), self-efficacy (16/22 agree or strongly agree, 2/22 somewhat agree), and adherence (12/22 agree or strongly agree, 6/22 somewhat agree). General themes for positive aspects of the program included increased patient confidence, decreased patient/family anxiety, and increased confidence that patients will take medication safely and correctly after discharge. General themes for the negative aspect of the program included increased workload and the desire for increased training on the SMP.

## Discussion

SMPs have been evaluated in patient populations in both the acute care and rehabilitation settings. To our knowledge, this is the first study that evaluated the efficacy and safety of a patient SMP in the allo-HSCT population. The results of our study demonstrated that the SMP was associated with better medication knowledge at discharge and at follow-up when compared to the previous standard of care. Notably, there was a significantly higher number of participants who completed post-secondary education or above in the pre-SMP group than in the SMP group (81% vs. 52%, *p*  =  0.029). As this result had the potential to skew higher medication knowledge scores in favor of the pre-SMP group, we feel that this strengthens our efficacy results. In addition, while not statistically significant our results did demonstrate that the SMP was associated with a trend towards improved patient-reported self-efficacy and reduced hospital readmissions.

### Efficacy endpoints: Knowledge, self-efficacy, adherence

While overall medication knowledge scores improved, participants in the SMP group actually fared worse on knowledge questions about medication side effects. While the clinical significance of this result is not known, it can be argued that knowledge of medication side effects plays a minimal role, if any, in medication adherence in our study population. Recipients of an allo-HSCT are closely monitored for clinical scenarios including GVHD, graft failure, and relapse of their primary disease, and are encouraged to report any new symptoms to their medical team. Additionally, recipients of an allo-HSCT take numerous medications, many of which have overlapping side effect profiles. Additionally, side effects of some of these medications could present similarly to symptoms of clinical events such as GVHD and infections. This makes it difficult for patients to keep track of which side effects can be attributed to which medication. Ultimately, several participants indicated verbally that they would be inclined to report any new sign or symptom that they experienced to the medical team, no matter the cause.

Our results found that the SMP did not result in a statistically significant difference in medication self-efficacy or patient-reported medication adherence. This result is similar to that of a randomized controlled trial in which the implementation of an SMP did not affect patient morale or ability to self-medicate on discharge.^3^ However, our study demonstrated that the SMP was associated with a trend towards improved self-efficacy at both discharge and follow-up. It is possible that with larger sample size, this result could be strengthened.

### Safety endpoints: Medication events and incidents, hospital readmissions

A previous study found that medication errors at a 1-month follow-up were significantly decreased after implementation of an SMP.^3^ In our study, 7 out of 31 participants (23%) experienced at least 1 medication event while participating in the SMP. While none of these events reached the patient, it is reasonable to hypothesize that these events would not have been caught and corrected should the patient have been at home. Had they been experienced after discharge, these events could have led to adverse events and/or hospital readmission.

Finally, our study found that readmission rates between 3–5 weeks post-discharge were 26% in the pre-SMP group versus 19% in the SMP group (*p*  =  0.54), which is consistent with the 30-day readmission rates post-allo-HSCT previously reported in the literature (17.5–39%).^13–15^ The wide range of readmission rates reported in the literature could be the result of differences in intensity of conditioning regimens, post-HSCT follow-up, and threshold to readmit between centers.^13–15^ Review of the electronic medical records after discharge revealed poor oral intake, GVHD, documented infection, and febrile neutropenia of unknown sources to be the most frequent causes of readmission in both groups. These results are similar to those of other studies describing the nature of hospital readmissions post-allo-HSCT.^13–15^ At this time, it is difficult to draw any conclusions regarding whether or not the SMP had an effect on the readmission rate.

### Patient and staff acceptability

Results from the patient and staff satisfaction surveys were positive. Patients stated that participating in the program led to increased confidence in taking their medications as well as increased preparedness for transition to home, and most indicated that they were grateful for the chance to practice managing their medications while still in the hospital. Staff members felt that the SMP was associated with increased confidence and decreased anxiety for patients and their families, as well as increased peace of mind for staff members. Negative aspects of the SMP identified by the patient and staff surveys were discussed amongst study team members (which include unit pharmacists) and, where possible, feedback informed improvement in practice. Of note, while there was feedback regarding the method of packaging medications for use in the SMP, medications are required to continue to be provided to patients unit dosed and individually labeled, rather than loose with a single label affixed to the vial or blister packed, as per hospital standards.

### Limitations

Our study had some limitations. First, the accuracy of self-efficacy and adherence results are dependent on the accuracy of patient-reported data. Second, an objective measure of medication adherence was not employed. In our study, medication self-efficacy was used as a surrogate indicator of medication adherence both at discharge and at follow-up, along with patient self-reported medication adherence via visual analog scale at follow-up. Studies have shown that subjective self-reporting, ideally with the use of a validated scale, is an ideal method of measuring medication adherence as it is easy to implement, is not costly, and allows for more context surrounding instances of nonadherence.^16^ Furthermore, objective measures such as pill counts or measured blood or urine drug levels are, while more concrete, not without their drawbacks.^16,17^ Pill counts inform whether or not the medication is present but give no information as to whether the correct dose was taken at the correct time. Measuring drug levels in blood or urine is invasive and can be confounded by factors such as renal function, hepatic function, fluid balance, acute illness, or drug interactions. Self-efficacy, the confidence one has in their ability to perform a task, has been shown to be a determinant of medication adherence.^11^ Our questionnaires included a modified version of a validated scale, SEAMS, used to measure medication self-efficacy. Additionally, visual analog rating scales have been shown to be more accurate than self-reported recall of specific missed medication incidents in estimating patient adherence, and in one study were shown to provide estimates of adherence that matched adherence rates obtained by unannounced pill counts.^18^

Another limitation to our study was its relatively small sample size. In order to maintain feasibility for our study, we chose to employ a sample size of convenience when enrolling participants. Additionally, study team members did not have control over follow-up appointment scheduling, cancellation, or unplanned weekend discharges. Previous studies evaluating an SMP in various patient populations had similarly small sample sizes ranging from approximately 10–100 participants.^3,4,6^

Finally, the patient satisfaction surveys were conducted on a cohort of patients different than those who completed the medication knowledge and self-efficacy questionnaire. The addition of patient and staff surveys to the study was part of a protocol amendment submitted in August 2019, well after initial data collection was completed. As original study participants had not provided consent to be contacted for any additional surveys or data collection, the decision was made to survey a different cohort of patients who had participated in the SMP during their hospital stay.

## Conclusion

The results of our study demonstrate that the SMP is associated with durable, improved medication knowledge at discharge and at 3–5 weeks post-discharge when compared to the previous standard of care. Our results also demonstrated that the SMP was associated with a trend towards improved patient-reported self-efficacy and reduced hospital readmissions. We hypothesize that medication events observed while patients were participating in the SMP could have led to adverse events and/or hospital readmissions if experienced after discharge. Finally, our survey results demonstrated that the SMP is associated with largely positive perceptions among both staff and patient-participants.

## Supplemental Material

sj-docx-2-opp-10.1177_10781552211043525 - Supplemental material for Evaluation of a patient self-medication program in allogeneic hematopoietic stem cell transplantation Click here for additional data file. Supplemental material, sj-docx-2-opp-10.1177_10781552211043525 for Evaluation of a patient self-medication program in allogeneic hematopoietic stem cell transplantation by Samantha Polito, Lina Ho, Ian Pang, Celina Dara and Auro Viswabandya in Journal of Oncology Pharmacy Practice
