# Supplementary material for: Evaluation of a patient self-medication program in allogeneic hematopoietic stem cell transplantation
Source: J Oncol Pharm Pract. 2021 Sep 27;28(8):1790–7. doi: 10.1177/10781552211043525 (PMC9623336; doi:10.1177/10781552211043525)
Supplement: sj-docx-2-opp-10.1177_10781552211043525 - Supplemental material for Evaluation of a patient self-medication program in allogeneic hematopoietic stem cell transplantation [file sj-docx-2-opp-10.1177_10781552211043525.docx]

# APPENDICES

*List of Appendices*

| **Appendix 1** | Medication knowledge and self-efficacy questionnaire 1 |
| --- | --- |
| **Appendix 2** | Medication knowledge and self-efficacy questionnaire 2 |
| **Appendix 3** | Self-Efficacy for Appropriate Medication Use Scale (SEAMS) |
| **Appendix 4** | Patient satisfaction survey |
| **Appendix 5** | Staff satisfaction survey |
| **Appendix 6** | Additional Tables and Figures |

## **APPENDIX 1: MEDICATION-TAKING KNOWLEDGE AND SELF-EFFICACY QUESTIONNAIRE 1**

**Date: __________ Participant Identification Number: ______**

**SECTION ONE- EDUCATION:**

What is the highest level of schooling that you have completed?

- Grade school (Grades 1 to 8)
- High school
- College
- University
- Professional Degree

**SECTION TWO- MEDICATION KNOWLEDGE:**

Please choose the best answer to the following 10 questions.

1. Why do you take cyclosporine / tacrolimus?
   1. To prevent Graft Versus Host Disease (GVHD)
   2. To prevent infection
   3. To increase iron levels
   4. To treat pain
2. What should you avoid while taking cyclosporine / tacrolimus?
   1. Orange juice (all types)
   2. Grapefruit juice
   3. Dairy products
   4. Meat products
3. On clinic days, when should you take your morning dose of cyclosporine / tacrolimus?
   1. At the regular scheduled time
   2. You should skip this dose entirely
   3. After your blood has been drawn
   4. In the late afternoon after lunch
4. What do you do if you forget to take your morning dose of cyclosporine / tacrolimus until right before dinner?
   1. Take the missed dose immediately, and then take the next dose at bedtime
   2. Skip this dose, and take double the amount at bedtime
   3. Skip this dose, and take the regular amount at bedtime
   4. Take the missed dose immediately, and do not take the bedtime dose
5. How many tablets of posaconazole should you take with each dose?
   1. One (1)
   2. Two (2)
   3. Three (3)
   4. Four (4)
6. Why do you take acyclovir / valganciclovir?
   1. To prevent bacterial infection
   2. To prevent fungal infection
   3. To prevent Graft Versus Host Disease (GVHD)
   4. To prevent viral infections
7. How many times per day do you take acyclovir / valganciclovir?
   1. One (1) time
   2. Two (2) times
   3. Three (3) times
   4. Four (4) times
8. Why do you take / receive Sulfamethoxazole and trimethoprim (Septra) / Pentamidine?
   1. To prevent a lung infection called Pneumocystis jirovecii pneumonia (PJP)
   2. To treat bacterial infection
   3. To treat viral infection
   4. To treat low magnesium levels
9. How often do you take / receive a dose of Sulfamethoxazole and trimethoprim (Septra) / Pentamidine?
   1. Once per day
   2. Twice per week
   3. Three times per week
   4. Once every 4 weeks
10. What is a possible side effect of magnesium that is important to report to your transplant team?
    1. Cough
    2. Headache
    3. Diarrhea or loose stools
    4. Rash

**SECTION THREE- SELF-EFFICACY:**

The following questions will gather information about your medication self-efficacy; that is, how confident you are that you can take your medications the way that your doctor intended. Please choose the best response for each of the 13 questions below.

**The following question stem applies to questions 1 to 4:**

**How confident are you that you can:**

1. take all of your medications without getting them mixed up?

A: Not confident B: Somewhat confident C: Very confident

2. take the correct amount of all of your medications?

A: Not confident B: Somewhat confident C: Very confident

3. take all of your medications at the correct scheduled time?

A: Not confident B: Somewhat confident C: Very confident

4. take all of your medications according to the correct instructions?

A: Not confident B: Somewhat confident C: Very confident

**The following question stem applies to questions 5 to 13 below:**

**How confident are you that you can take your medications correctly when:**

5. the schedule to take the medications is not convenient?

A: Not confident B: Somewhat confident C: Very confident

6. no one reminds you to take the medications?

A: Not confident B: Somewhat confident C: Very confident

7. you are away from home?

A: Not confident B: Somewhat confident C: Very confident

8. you have a busy day planned?

A: Not confident B: Somewhat confident C: Very confident

9. they cause some side effects?

A: Not confident B: Somewhat confident C: Very confident

10. you are feeling sick (like having a cold or the flu)?

A: Not confident B: Somewhat confident C: Very confident

11. you are feeling fine?

A: Not confident B: Somewhat confident C: Very confident

12. you get a refill of your old medications and some of the pills look different than usual?

A: Not confident B: Somewhat confident C: Very confident

13. a doctor changes your medications?

A: Not confident B: Somewhat confident C: Very confident

Thank you for taking the time to complete this questionnaire.

## **APPENDIX 2: MEDICATION-TAKING KNOWLEDGE AND SELF-EFFICACY QUESTIONNAIRE 2**

**Date: __________ Participant Identification Number: ______**

**SECTION ONE- EDUCATION:**

What is the highest level of schooling that you have completed?

- Grade school (Grades 1 to 8)
- High school
- College
- University
- Professional Degree

**SECTION TWO- MEDICATION KNOWLEDGE:**

Please choose the best answer to the following 10 questions.

1. Why do you take cyclosporine / tacrolimus?
   1. To prevent Graft Versus Host Disease (GVHD)
   2. To prevent infection
   3. To increase iron levels
   4. To treat pain
2. What should you avoid while taking cyclosporine / tacrolimus?
   1. Orange juice (all types)
   2. Grapefruit juice
   3. Dairy products
   4. Meat products
3. On clinic days, when should you take your morning dose of cyclosporine / tacrolimus?
   1. At the regular scheduled time
   2. You should skip this dose entirely
   3. After your blood has been drawn
   4. In the late afternoon after lunch
4. What do you do if you forget to take your morning dose of cyclosporine / tacrolimus until right before dinner?
   1. Take the missed dose immediately, and then take the next dose at bedtime
   2. Skip this dose, and take double the amount at bedtime
   3. Skip this dose, and take the regular amount at bedtime
   4. Take the missed dose immediately, and do not take the bedtime dose
5. How many tablets of posaconazole should you take with each dose?
   1. One (1)
   2. Two (2)
   3. Three (3)
   4. Four (4)
6. Why do you take acyclovir / valganciclovir?
   1. To prevent bacterial infection
   2. To prevent fungal infection
   3. To prevent Graft Versus Host Disease (GVHD)
   4. To prevent viral infections
7. How many times per day do you take acyclovir / valganciclovir?
   1. One (1) time
   2. Two (2) times
   3. Three (3) times
   4. Four (4) times
8. Why do you take / receive Sulfamethoxazole and trimethoprim (Septra) / Pentamidine?
   1. To prevent a lung infection called Pneumocystis jirovecii pneumonia (PJP)
   2. To treat bacterial infection
   3. To treat viral infection
   4. To treat low magnesium levels
9. How often do you take / receive a dose of Sulfamethoxazole and trimethoprim (Septra) / Pentamidine?
   1. Once per day
   2. Twice per week
   3. Three times per week
   4. Once every 4 weeks
10. What is a possible side effect of magnesium that is important to report to your transplant team?
    1. Cough
    2. Headache
    3. Diarrhea or loose stools
    4. Rash

**SECTION THREE- SELF-EFFICACY:**

The following questions will gather information about your medication self-efficacy; that is, how confident you are that you can take your medications the way that your doctor intended. Please choose the best response for each of the 13 questions below.

**The following question stem applies to questions 1 to 4:**

**How confident are you that you can:**

1. take all of your medications without getting them mixed up?

A: Not confident B: Somewhat confident C: Very confident

2. take the correct amount of all of your medications?

A: Not confident B: Somewhat confident C: Very confident

3. take all of your medications at the correct scheduled time?

A: Not confident B: Somewhat confident C: Very confident

4. take all of your medications according to the correct instructions?

A: Not confident B: Somewhat confident C: Very confident

**The following question stem applies to questions 5 to 13 below:**

**How confident are you that you can take your medications correctly when:**

5. the schedule to take the medications is not convenient?

A: Not confident B: Somewhat confident C: Very confident

6. no one reminds you to take the medications?

A: Not confident B: Somewhat confident C: Very confident

7. you are away from home?

A: Not confident B: Somewhat confident C: Very confident

8. you have a busy day planned?

A: Not confident B: Somewhat confident C: Very confident

9. they cause some side effects?

A: Not confident B: Somewhat confident C: Very confident

10. you are feeling sick (like having a cold or the flu)?

A: Not confident B: Somewhat confident C: Very confident

11. you are feeling fine?

A: Not confident B: Somewhat confident C: Very confident

12. you get a refill of your old medications and some of the pills look different than usual?

A: Not confident B: Somewhat confident C: Very confident

13. a doctor changes your medications?

A: Not confident B: Somewhat confident C: Very confident

**SECTION FOUR- MEDICATION ADHERENCE:**

Sometimes it is difficult to take all of your medications exactly how your doctor prescribed them. Thinking back over the past month, how much of your medications have you taken? For example, 0% means that you have not taken any of your medications, 50% means that you have taken half of your medications, and 100% means that you have taken all of your medications. Put a cross on the line below showing your best guess.


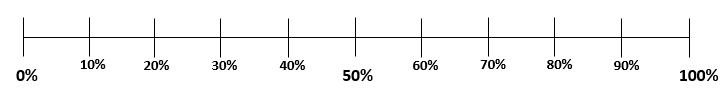


Thank you for taking the time to complete this questionnaire.

## **APPENDIX 3: UNMODIFIED 13-POINT SELF-EFFICACY FOR APPROPRIATE MEDICATION USE SCALE (SEAMS)**

Please answer each of the following questions using the rating scale below:

1 = not confident

2 = somewhat confident

3 = very confident

1. How confident are you that you can take your medications correctly when you take several different medicines each day?
2. How confident are you that you can take your medications correctly when you take medicines more than once a day?
3. How confident are you that you can take your medications correctly when you are away from home?
4. How confident are you that you can take your medications correctly when you have a busy day planned?
5. How confident are you that you can take your medications correctly when they cause some side effects?
6. How confident are you that you can take your medications correctly when no one reminds you to take the medicine?
7. How confident are you that you can take your medications correctly when the schedule to take the medicine is not convenient?
8. How confident are you that you can take your medications correctly when your normal routine gets messed up?
9. How confident are you that you can take your medications correctly when you are not sure how to take the medicine?
10. How confident are you that you can take your medications correctly when you are not sure what time of the day to take your medicine?
11. How confident are you that you can take your medications correctly when you are feeling sick (like having a cold or the flu)?
12. How confident are you that you can take your medications correctly when you get a refill of your old medicines and some of the pills look different than usual?
13. How confident are you that you can take your medications correctly when a doctor changes your medicines?

## **APPENDIX 4: PATIENT SATISFACTION SURVEY**

**Date: __________ Participant Identification Number: ______**

**Allogeneic Hematopoietic Stem Cell Transplant
Patient Self Medication Program**

**PATIENT SATISFACTION SURVEY**

Over the past year, the allogeneic hematopoietic stem cell transplantation (allo-HSCT) patient Self Medication Program (SMP) has been started at the **[REDACTED FOR BLINDING]**.

We are conducting a study to look at how well the SMP works, and what you think about it. This study is called “Evaluation of a Patient Self Medication Program in Allogeneic Hematopoietic Stem Cell Transplantation”. Because you participated in the SMP during your hospital admission, we are asking for your feedback.

This survey should take approximately 10 minutes to complete. Your responses will be confidential. Taking part in the survey is a decision that you will make. If you decide not to take part, the care you receive will not change. Completion of this survey will mean that you agree to take part in the study and that your answers will be used for research and making the SMP better.

The main investigator for this study is Dr. **[REDACTED FOR BLINDING]**.

If you have any questions about the study, you can contact the study coordinator **[REDACTED FOR BLINDING]**.

(Please note that communication via e-mail is only secure if you communicate from **[REDACTED FOR BLINDING]** to **[REDACTED FOR BLINDING]** email addresses.)

**Please circle your answer to the following questions. If you have more to say, please use the spaces below.**

1. Have you ever participated in a SMP before your recent allo-HSCT admission?

Yes No

If yes, please tell us about it:

______________________________________________________________________

______________________________________________________________________

1. The **amount** **of information** that you received about the SMP **before** **you started the program** was:


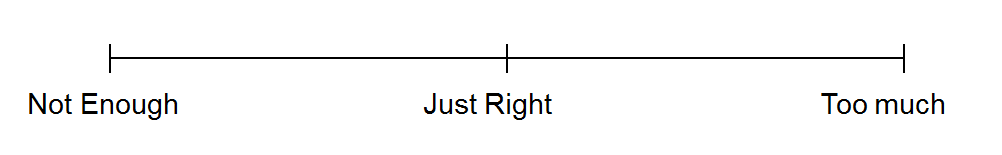


Please tell us how this could have been better:

______________________________________________________________________

______________________________________________________________________

1. The **amount** **of information** you received about the SMP **while you were in the program** was:


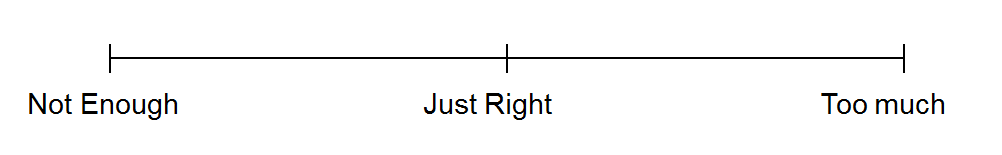


Please tell us how this could have been better:

______________________________________________________________________

______________________________________________________________________

1. The **language** used to explain the SMP to you was easy to understand.


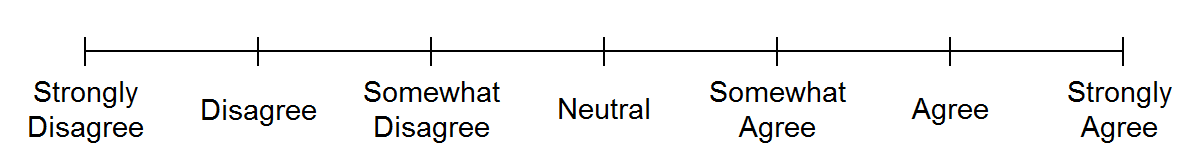


Please tell us how this could have been better:

______________________________________________________________________

______________________________________________________________________

1. The **instructions** for following the SMP were clear.


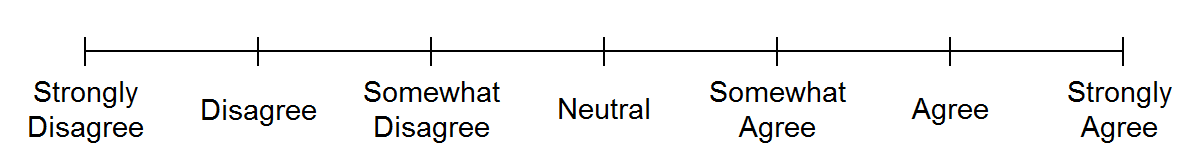


Please tell us how this could have been better:

______________________________________________________________________

______________________________________________________________________

1. The **drug chart (or schedule)** given to you was easy to follow.


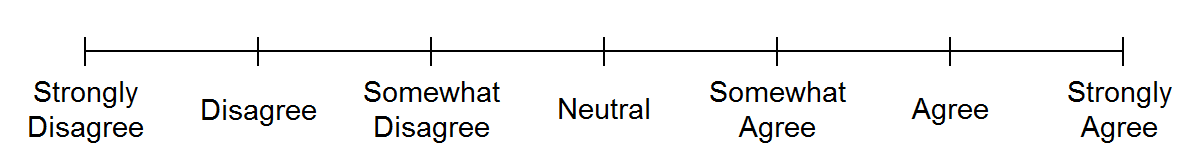


Please tell us how this could have been better:

______________________________________________________________________

______________________________________________________________________

1. You felt **safe** while you were participating in the SMP.


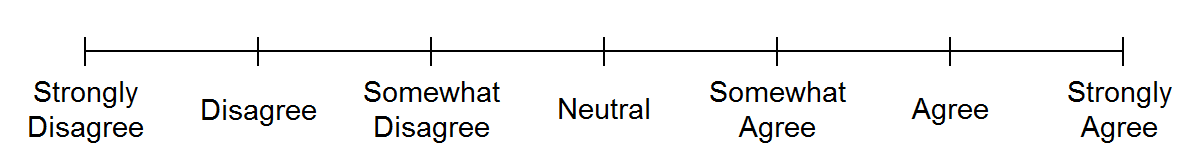


Please tell us why:

______________________________________________________________________

______________________________________________________________________

1. The SMP helped you to **know more** about your drugs.


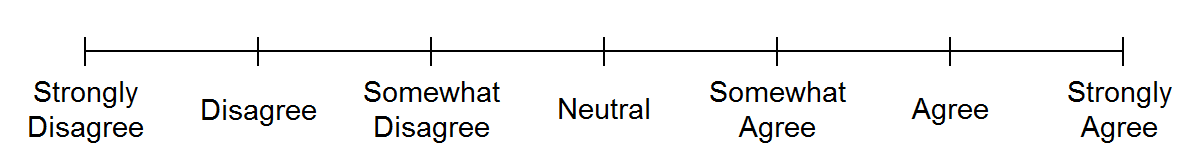


Please tell us why:

______________________________________________________________________

______________________________________________________________________

1. The SMP helped you to become **more sure** about how to take your drugs.


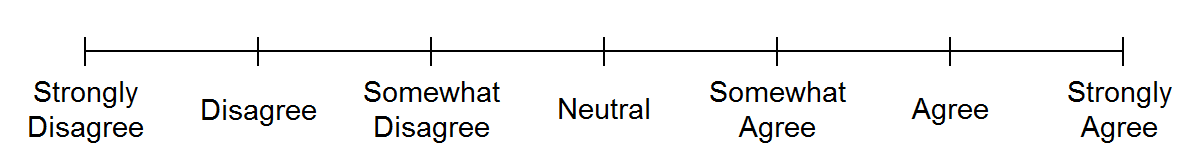


Please tell us why:

______________________________________________________________________

______________________________________________________________________

1. The SMP helped to prepare you be able to take your drugs correctly **even after** **the number of pills you had to take was changed**.


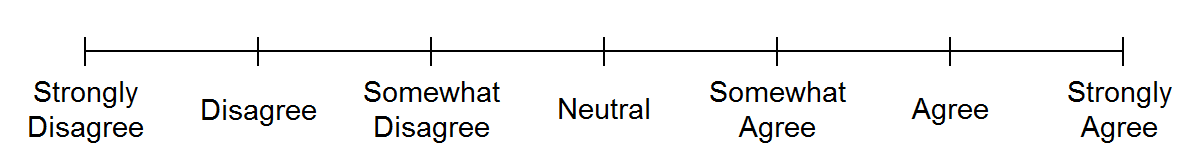


Please tell us why:

______________________________________________________________________

______________________________________________________________________

1. When you were home, did you need any help with your drugs?

Yes No

If yes, please tell us why:

______________________________________________________________________

______________________________________________________________________

1. What did you like about the SMP?

______________________________________________________________________

______________________________________________________________________

1. What about the SMP can be made better?

______________________________________________________________________

______________________________________________________________________

Thank you for your time.

## **APPENDIX 5: STAFF SATISFACTION SURVEY**

Note: Staff members were able to provide additional comments for questions #2-9.

1. Please indicate your profession:
   1. Physician
   2. Nurse Practitioner
   3. Nurse
   4. Pharmacist
   5. Student
   6. Other (please specify)
2. Do you have any prior experience with a patient SMP?
3. Are you satisfied with the training that you received prior to the implementation of the allo-HSCT patient SMP?
   1. Very dissatisfied
   2. Dissatisfied
   3. Somewhat dissatisfied
   4. Neutral
   5. Somewhat satisfied
   6. Satisfied
   7. Very satisfied
4. Do you feel that your workload has increased as a result of the allo-HSCT patient SMP?
   1. Yes
   2. No
5. Have you experienced any difficulties related to the allo-HSCT patient SMP?
   1. Yes (please specify)
   2. No
6. The allo-HSCT patient SMP has a positive impact on patients’ medication knowledge.
   1. Strongly disagree
   2. Disagree
   3. Somewhat disagree
   4. Neutral
   5. Somewhat agree
   6. Agree
   7. Strongly agree
7. The allo-HSCT patient SMP has a positive impact on patients’ confidence in medication management.
   1. Strongly disagree
   2. Disagree
   3. Somewhat disagree
   4. Neutral
   5. Somewhat agree
   6. Agree
   7. Strongly agree
8. The allo-HSCT patient SMP has a positive impact on patients’ medication adherence.
   1. Strongly disagree
   2. Disagree
   3. Somewhat disagree
   4. Neutral
   5. Somewhat agree
   6. Agree
   7. Strongly agree
9. Do you have any concerns about the allo-HSCT patient SMP?
   1. Yes (please specify)
   2. No
10. Other comments/suggestions?

## **APPENDIX 6: ADDITIONAL TABLES AND FIGURES**

| **Appendix 6A** | Distribution of Incorrect Answers to Knowledge-Based Questions |
| --- | --- |
| **Appendix 6B** | Distribution of Answers to Self-Efficacy Questions |

**APPENDIX 6A**

**Table 1a: Distribution of incorrect answers to knowledge-based questions, Pre-SMP group**

|  | **Area of medication knowledge** | | | | | | | | | |
| --- | --- | --- | --- | --- | --- | --- | --- | --- | --- | --- |
|  | **Indication** | | | **How and when to take it** | | | | | **Important**  **ADRs*** | **What to do if dose missed** |
| **Question Number** | #1 | #6 | #8 | #2 | #3 | #5 | #7 | #9 | #10 | #4 |
| **Discharge**  **n (%)** | 0 (0) | 7 (19) | 3 (8) | 0 (0) | 1 (3) | 3 (8) | 6 (16) | 4 (11) | 5 (13) | 8 (22) |
| **Follow-Up**  **n (%)** | 0 (0) | 9 (26) | 3 (9) | 0 (0) | 1 (3) | 6 (18) | 2 (6) | 4 (12) | 8 (23) | 1 (3) |

*ADRs = adverse drug reactions

**Table 1b: Distribution of incorrect answers to knowledge-based questions, SMP group**

|  | **Area of medication knowledge** | | | | | | | | | |
| --- | --- | --- | --- | --- | --- | --- | --- | --- | --- | --- |
|  | **Indication** | | | **How and when to take it** | | | | | **Important**  **ADRs*** | **What to do if dose missed** |
| **Question Number** | #1 | #6 | #8 | #2 | #3 | #5 | #7 | #9 | #10 | #4 |
| **Discharge**  **n (%)** | 2 (9) | 4 (18) | 2 (9) | 1 (5) | 0 (0) | 4 (18) | 0 (0) | 2 (9) | 5 (23) | 2 (9) |
| **Follow-Up**  **n (%)** | 3 (21) | 2 (14) | 1 (7) | 1 (7) | 0 (0) | 0 (0) | 3 (21) | 0 (0) | 4 (29) | 0 (0) |

*ADRs = adverse drug reactions

**APPENDIX 6B**

**Figure 1a: Distribution of answers to self-efficacy questions at discharge, Pre-SMP group**

**Figure 1b: Distribution of answers to self-efficacy questions at follow-up, Pre-SMP group**

**Figure 2a: Distribution of answers to self-efficacy questions at discharge, SMP group**

**Figure 2b: Distribution of answers to self-efficacy questions at follow-up, SMP group**
